# Multidisciplinary Therapeutic Management in Complex Cervical Trauma

**DOI:** 10.3390/medicina59030596

**Published:** 2023-03-17

**Authors:** Florentina Severin, Andrei-Mihail Rosu, Mirela Tiglis, Laura-Elisabeta Checherita, Gina Stegaru, Mihail Dan Cobzeanu, Razvan Hainarosie, Bogdan Mihail Cobzeanu, Octavian Dragos Palade

**Affiliations:** 1 Surgery Department, University of Medicine and Pharmacy “Grigore T. Popa”, 700115 Iasi, Romania; florentina-s-severin@umfiasi.ro (F.S.);; 2 Department of Anesthesia and Intensive Care, Emergency Clinical Hospital of Bucharest, 014461 Bucharest, Romania; 3 Surgery Department, University of Medicine and Pharmacy “Carol Davila”, 37 Dionisie Lupu Str., 020021 Bucharest, Romania

**Keywords:** otorhinolaryngology, surgery, multidisciplinary treatment, complex cervical trauma

## Abstract

*Background and Objectives*: In the current literature, mandatory surgical exploration is a controversial topic, with some advocating for it and others against it, proposing a selective conservative management. This multidisciplinary therapeutic approach is based on clinical examination and serial paraclinical explorations associated with supportive drug treatment. *Materials and Methods*: The study group consisted of 103 patients with complex cervical trauma pathology produced by various mechanisms such as car or domestic accidents, aggression, ballistic trauma, self-inflicted attempts, hanging or strangulation hospitalized in the Ear, Nose and Throat (E.N.T.) Clinic, at “St. Spiridon” Iași Hospital, between 2012 and 2016. *Results*: The universal clinical indication for urgent surgical exploration of the patient with complex cervical trauma is the presence of the following symptoms: unstable vital signs, significant pulsatile bleeding, hematoma with a substantial increase in size, shock, airway obstruction, open airway wound, hematemesis, or hemoptysis. In this context, we considered it worthwhile to research the management of complex cervical trauma in a reference university medical center, alongside the analysis of the patient’s characteristics under different aspects (demographic, pathological aspects, therapeutic). *Conclusions*: Complex cervical trauma has a variety of clinical aspects, with a variable evolution, which involves multidisciplinary therapeutic management. The increasing trauma rate is one of the main public health problems, requiring epidemiological studies, and the implementation of control strategies.

## 1. Introduction

The management of cervical trauma has been a topic of great interest and controversy over the years. The need for surgical exploration of all cervical wounds’ dates back to the time of the Second World War (WWII). However, the evolution of the emergency medical system and paraclinical investigations has contributed to the improvement of global statistics regarding the mortality and morbidity of this pathology.

Currently, studies reported by various authors show similar mortality and morbidity regarding the approach of clinical surveillance and non-exploratory diagnosis in selected cases versus surgical management for all patients. The universal clinical indication for urgent surgical exploration of the patient with complex cervical trauma is the presence of the following symptoms: unstable vital signs, significant pulsatile bleeding, hematoma with a substantial increase in size, shock, airway obstruction, open airway wound, hematemesis or hemoptysis [1]. Proponents of mandatory surgical management argue that any injury that penetrates the platysma should be explored in the operating room. In particular, zone II is mainly due to the rich content of critical neurovascular structures.

On the other hand, in the last five years, the current otolaryngology opinion issues the hypothesis, with already quite a few followers, in favor of selective surgical management. Paraclinical examinations are required, which include angiography, esophagography, panendoscopy, and computed tomography. Explorations are indicated only in hemodynamically and respiratory stable patients. This management strategy is preferably performed only in hospital facilities with appropriate logistics [2].

We present a retrospective and prospective study of complex cervical trauma pathology secondary to various accidents or aggressions, which required surgical interventions. Our main objectives were to analyze the management of complex cervical trauma in a reference university medical center, and the analysis of the patient’s characteristics under different aspects (demographic, pathological aspects, therapeutic).

## 2. Materials and Methods

The study group was made up of 103 patients admitted to the Ear, Nose and Throat (E.N.T.) Clinic, “St. Spiridon”, Iasi, between 2012–2016, with complex cervical trauma pathology produced by various mechanisms, such as car accidents, domestic accidents, assaults, ballistic trauma, self-inflicted attempts, hanging or strangulation. However, this study was partially limited by the fact that information on particular patients was incomplete, due to the retrospective nature of the research and the lack of uniformity regarding the description of the operative technique, along with the results of the paraclinical investigations performed, as there was no existing standardized protocol; a situation that could bring some damage to the data identified on this study. The study was started in 2014 and it consisted of two parts. The patients included in the retrospective study were those admitted to the ENT clinic of “Saint Spiridon” Hospital, Iasi, in the period 2012–2014. Likewise, the patients included the prospective study were those patients admitted to the ENT Clinic in during 2014–2016. We defined the complex cervical trauma as the lesions that are penetrating platysma and involve at least the superior aero-digestive tract, vascular, neurologic, thyroidian or salivary gland structures.

The data were centralized in an SPSS 18.0 database and processed with the statistical functions to which they lend themselves at the significance threshold of 95%. Using specific statistical methods, it was possible to calculate the mean value and the standard deviation (SD); quantitative variables were compared using the Student’s t-test, and the Chi-square test assessed quantitative parameters. The ANOVA test was used to evaluate descriptive statistical indicators: minimum, maximum, mean, median, standard deviation, standard error of the mean, and variance. The Skewness, Kurtosis (−2 < *p* < 2) method tests the normality of the series of values. In calculating the significant difference between the two means, the Student’s t-test considers the measurement of variability and the weight of the observations. F-test (ANOVA) was used to compare values with normal distributions in three or more groups. The Pearson correlation coefficient was used to establish the existence of correlations, and their intensity between various numerical variables studied. The type of correlation was expressed by the sign of the Pearson correlation coefficient, and the power of the link between the variables was represented by its value. Statistical significance was set at the *p* < 0.05 threshold for a 95% confidence interval. Univariate and multivariate analysis by logistic regression was used to determine the variables correlated with the presence or absence of the studied events, identifying statistically significant independent parameters.

General inclusion criteria had in view the information selected from the observation sheets and fell into the following categories: demographics data, epidemiological characteristics, lesion appearance and mechanism, location cervical corresponding to the defined areas of the neck, the type and extent of the damaged tissues, paraclinical investigations carried out, associated pathologies or relapses, therapeutic approach, complications along with the data obtained from the periodic consultations performed upon discharge from the hospital.

General exclusion criteria were under 18 years of age, the patient’s refusal to participate, the presence of previous cervical trauma, but without pathological lesions or only superficial lesions without being accompanied by the above-mentioned symptoms.

## 3. Results

The patients selected for this study presented with closed and open penetrating cervical traumatic injuries caused by self-inflicted, interpersonal aggression and accidental mechanism. The symptoms and clinical signs identified varied according to the location of the injury and the affected visceral or extra-visceral structure, and the concomitant existence of other traumas with other sites. Dysphonia, dyspnea, various degrees of acute respiratory failure, cervical subcutaneous emphysema, and a mid-cervical blowing wound are characteristic elements of the involvement of the laryngotracheal axis. Pharyngoesophageal damage is characterized by dysphagia and subcutaneous emphysema. A descending injury from zone I is associated with acute respiratory distress, pneumothorax or hemothorax, signs of cardiac tamponade suggesting damage to the apex of the lung and the vessels at the base of the neck, nerve involvement of the cervical plexus or cranial nerves, which may decrease the sensitivity and motility of the superior limbs. In parallel with the clinical examination, respiratory and cardiac vital signs were monitored, blood samples were taken, and the indication was established regarding paraclinical investigations or the need for emergency surgical exploration depending on the stability of Surgical explorations.

In the study group, made up of 103 patients hospitalized in the ENT clinic, the age varied between 17 and 78 years, the average age was 43 years (standard deviation = 15.67), registering a slightly higher average value in the male sex (43.41 vs. 42.09 years; *p* = 0.793). patients come more frequently from rural areas (56.6%), they are predominantly male (89.6%), under the age of 45 (55.7%); the lesional determining mechanism is noted more frequently through aggression (39.6%), followed by the self-inflicted mechanism (39.6%) and the accidental mechanism (22.6%).

**Figure 1:**
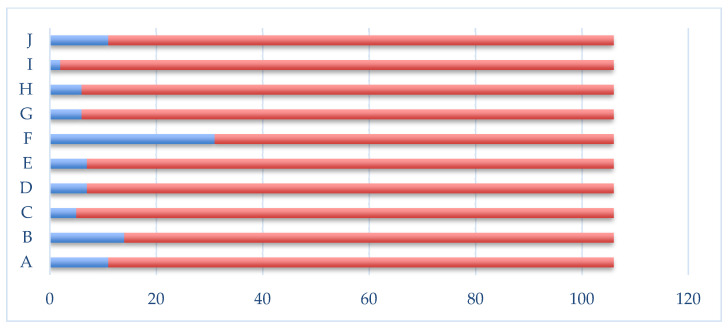
Distribution of complex aero-digestive cervical trauma cases and associated injuries. A—section of the thyrohyoid membrane with the interest of the hypopharynx. B—fracture of the laryngeal cartilages with laceration of the epiglottis, vocal cords, and pyriform sinuses. C—dilacerations with retrocricoid hematomas. D—sectioning of the cricothyroid membrane and partial or total involvement of the cricoid cartilage. E—sectioning of the crico-tracheal membrane and the tracheal rings. F—dissection of the superficial jugular veins. G—internal jugular vein involvement. H—thyroid gland interest and I—the interest of the submandibular gland. J—Polytraumatism.

In this study, the cases that required surgical intervention were characterized by complex aero-digestive, thyroid, vascular, and polytrauma injuries. Thus, the following were identified: section of the thyrohyoid membrane with involvement of the hypopharynx (11 cases), fracture of the laryngeal cartilages with dilaceration of the epiglottis, vocal cords and pyriform sinuses (14 cases), dilacerations with retrocricoid hematomas (5 cases), sectioning of the cricothyroid membrane and interest partial or total cricoid cartilage (7 cases), sectioning of the crico-tracheal membrane and tracheal rings (7 cases), sectioning of the superficial jugular veins (31 cases), involvement of the internal jugular vein (6 cases), involvement of the thyroid gland (6 cases), involvement of the submandibular gland (5 cases), polytraumas due to traffic accidents or falls from a height with complex thoracic, abdominal, craniofacial and limb injuries (11 cases) (Figure 1).

**Figure 2:**
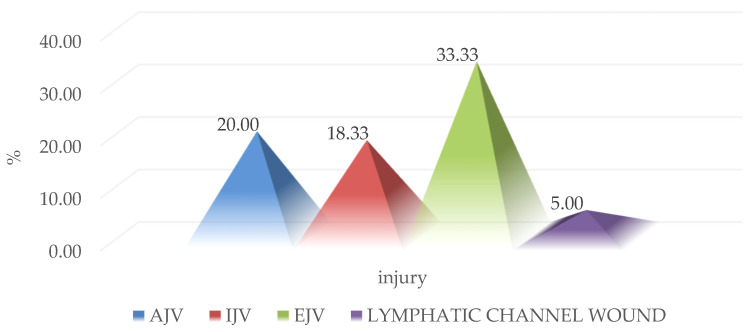
Distribution of venous lesions that required surgical treatment. (AJV = anterior jugular vein, IVJ = internal jugular vein, EJV = external jugular vein).

From the total number of vascular lesions of the anterior, external, and internal jugular veins, the following lesions were identified: contusions, adventitial lesions, and complete or partial sections, to which is added a case of penetrating wound involving the thoracic duct. These injuries required parietal reconstruction and vascular ligature to perform hemostasis, depending on the case, through specific techniques (Figure 2).

**Figure 3:**
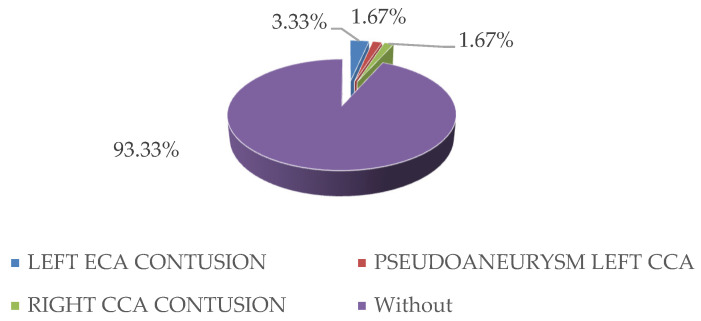
Distribution and characteristics of carotid lesions that required specific surgical treatment. (ECA = external carotid artery, CCA = common carotid artery).

Regarding the arterial lesions of the carotid system, adventitious contusion-type lesions of the common and external carotid arteries and pseudoaneurysm-type lesions at the level of the common carotid artery were identified in our study (Figure 3).

**Figure 4:**
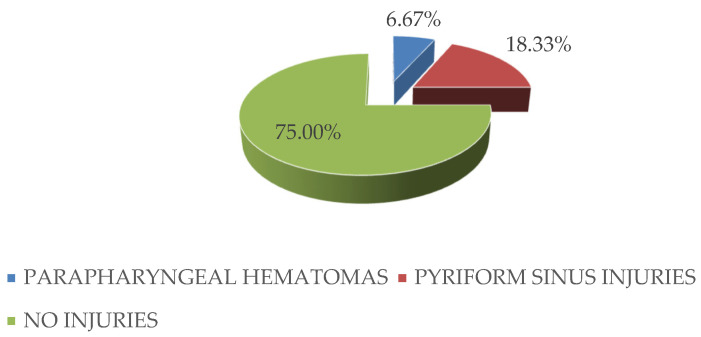
Distribution and characteristics of hypopharyngeal and parapharyngeal lesions that required surgical therapeutic management.

As for the penetrating hypopharyngeal lesions, in the entire study group, penetrating lesions of the pharyngeal wall were identified only at the level of the piriform sinuses with the development of parapharyngeal hematomas that had a posterior prevertebral extension and determined compressive phenomena with the onset of acute upper respiratory insufficiency and dysphagia (Figure 4).

**Figure 5:**
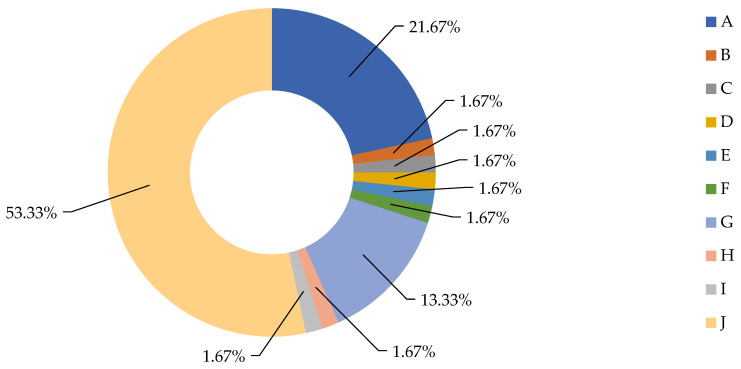
Distribution and characteristics of traumatic laryngeal injuries that required a surgical therapeutic approach. A—vocal cord injuries. B—arytenoid cartilage injuries. C—cricoid cartilage fracture. D—cricotracheal membrane section. E—section thyroyidian membrane. F—paralaryngian hematoma. G—total or partial thyroid cartilage fractures. H—section thyrohyoid membrane, epiglottic exposure, and laryngeal continuity solution. I—continuity solution at the laryngeal level with the appearance of cervical emphysema. J—no laryngeal injuries.

The appearance of traumatic laryngeal lesions identified both clinically, paraclinical, and intraoperatively is very diverse, including lesions of the mucosa, vocal cords, laryngeal cartilages, thyrohyoid and cricothyroid membrane, as illustrated in Figure 5 and on which specific therapeutic intervention was performed, both surgically and medically.

**Figure 6:**
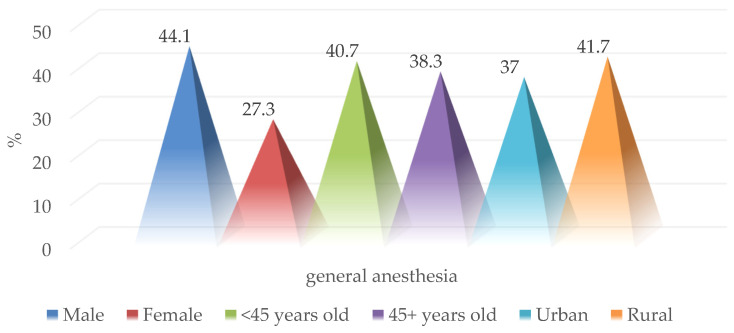
Epidemiological characteristics of patients with surgical exploration performed under general anesthesia.

Surgical exploration under general anesthesia was performed in 42 of the 103 patients in the study group (39.6%), more frequently in males (44.1% vs. 27.3%; *p* = 0.656), age group under 45 years (40.7% vs. 38.3%; *p* = 0.803) and with a rural background (37% vs. 41.7%; *p* = 0.623) (Figure 6).

**Figure 7:**
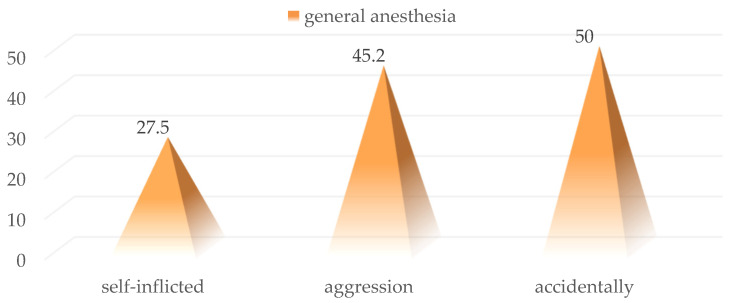
Distribution of cases surgically explored under general anesthesia according to the injury mechanism.

Regarding the mechanism of production of traumatic injuries, the statistical analysis shows that the use of surgical exploration through general anesthesia was more common in patients with penetrating injuries produced by aggression and accidentally (27.5% vs. 45.2% and 50%; *p* = 0.05) (Figure 7).

**Figure 8:**
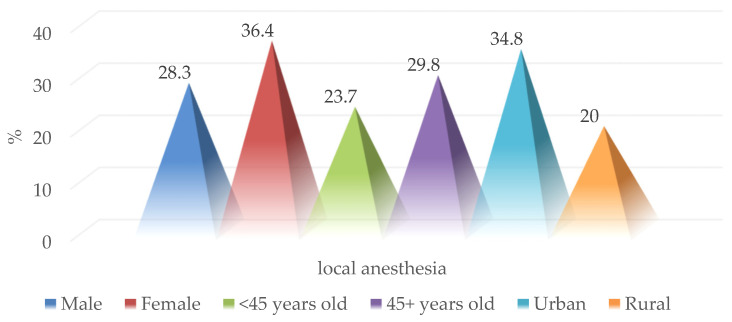
Epidemiological characteristics of patients with surgical exploration performed under local anesthesia.

Surgical exploration of penetrating wounds under local anesthesia was performed in 28 of the patients in the study group (26.4%), more frequently in females (36.4% vs. 28.3%; *p* = 0.443), age group over 45 years (23.7% vs. 29.8%; *p* = 0.483) and the urban environment (34.8% vs. 20%; *p* = 0.688) (Figure 8).

**Figure 9:**
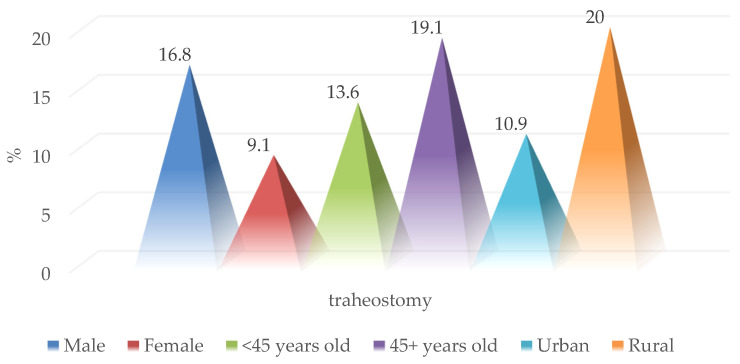
Epidemiological characteristics of patients who underwent tracheostomy.

**Figure 10:**
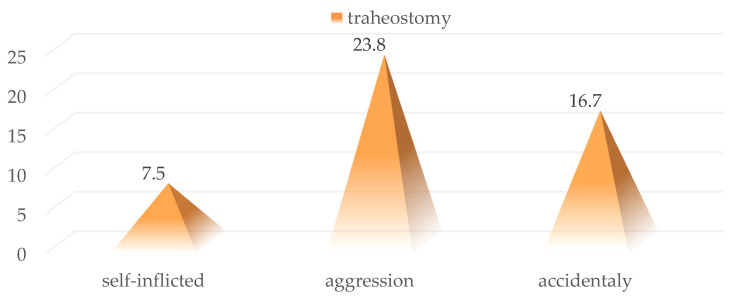
Distribution of cases in the batch in which tracheostomy was performed according to the lesion mechanism.

Tracheostomy was performed in 17 patients included in the study (16%) without significant differences between sexes, age groups, or place of residence (Figure 9). In addition, the need to secure the airway by performing a tracheostomy was not statistically significantly correlated with a specific injury mechanism determining cervical trauma (7.5% vs. 23.8% and 16.7%; *p* = 0.117) (Figure 10).

**Figure 11:**
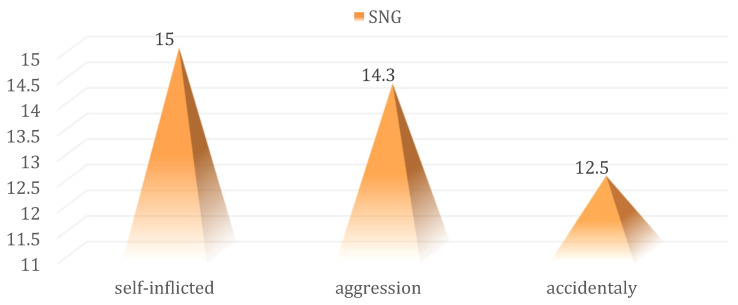
Distribution of cases in the batch to which SNG was applied according to the lesion mechanism.

Complex penetrating traumatic injuries of the aero-digestive tract require local digestive rest and the application of a nasogastric tube. However, the recommendation to apply the nasogastric tube was not statistically significantly correlated with the lesion mechanism, which produced the penetrating traumatic injury (15% vs. 14.3% and 12.5%; *p* = 0.961) (Figure 11).

**Figure 12:**
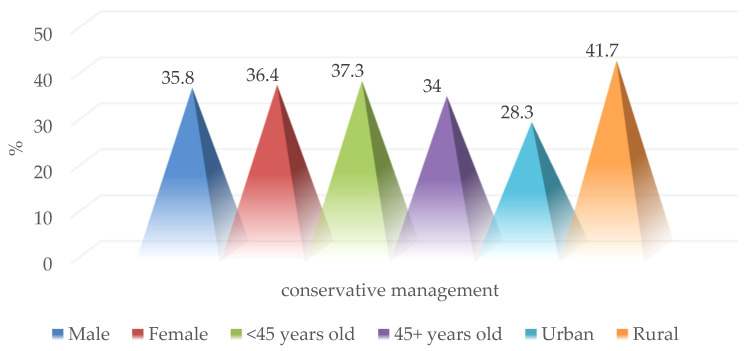
Epidemiological characteristics of patients with conservative management.

Conservative therapeutic management, consisting of observation, serial clinical examination, paraclinical explorations, and supportive drug treatment, was carried out in 38 of the selected patients (35.8%) without significant differences between sexes, age groups, or the place of residence (Figure 12).

**Figure 13:**
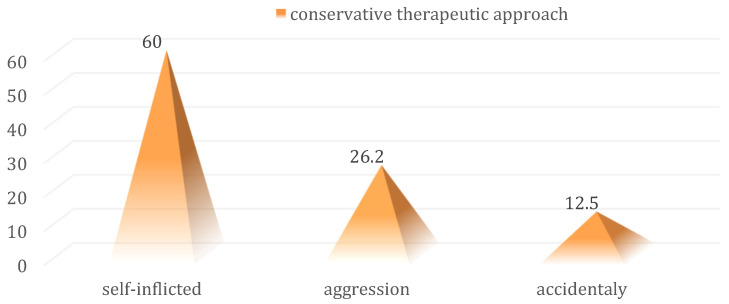
Distribution of cases in the batch with indications for conservative management according to the lesion mechanism.

The conservative therapeutic approach correlated statistically significantly with the self-inflicted mechanism (60% vs. 26.2% and 12.5%; *p* = 0.001) (Figure 13).

**Figure 14:**
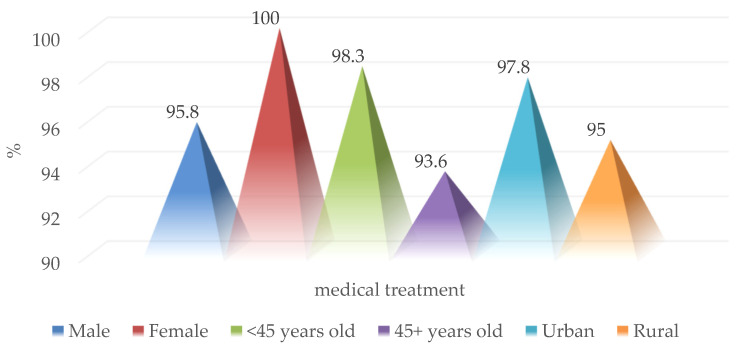
Epidemiological characteristics of patients who received medical treatment.

The need for the administration of supportive drug treatment was identified in 102 patients from the group (96.2%), without significant differences between sexes, age groups, or the place of residence (Figure 14).

Postoperative complications were noted in 6 patients (5.7%), all male, five aged over 45 years, and five from rural areas. The postoperative complications identified were the following: partial dehiscence of the postoperative wound, parotid salivary fistula, postoperative wound infection with Enterobacter, stroke, pneumococcal pneumonia, post-traumatic left genial edema, dysphonia, and torticollis.

## 4. Discussion

The essential therapeutic attitude in complex cervical trauma is ensuring the airways’ safety through orotracheal intubation or emergency tracheostomy and hemostasis control, followed by clinical and paraclinical investigations, then exploration and reconstructive surgical treatment. In cases where the hemodynamically unstable patient could not be investigated paraclinical and presented traumatic injuries with a penetrating character, urgent surgical exploration is required. This exploration can be performed under general anesthesia with orotracheal intubation, tracheostomy, or local anesthesia.

The importance of first aid, as well as emergency therapeutic measures in other health facilities, must include primary hemostasis and the proper support of cardio-respiratory parameters. Transport must be ensured in safe conditions and as soon as possible, considering the complications that can occur in the first hours after the trauma. In emergencies, the airway was secured (freeing the oral cavity of secretions and blood, possibly intubating the patient or tracheotomy), immobilizing the cervical spine, accessing the venous lines, and obtaining a correct anamnesis regarding the circumstances of the accident, the associated diseases, the state of consciousness. Intubation through the cervical area is indicated when there are injuries to the oral cavity, pharynx, or larynx. Orotracheal intubation can accentuate pharyngolaryngeal lesions, causing the extension of some lesions from the level of the piriform sinus to the mediastinum. In lesions with significant cervical tissue destruction, intubation was performed through the continuity solution at the level of the sectioned thyrohyoid membrane.

According to the European Manual of Medicine, Otorhinolaryngology, Head and Neck Surgery, the standard of investigation and exploration procedures in the case of cervical traumatology is applied depending on the presence or not of the hemodynamic and neurological stability criteria. The unstable patient will be provided with an airway simultaneously with the treatment of shock and cervical surgical exploration; postoperative achievement of hemodynamic stability and neurological will allow additional endoscopic and imaging investigations to be performed. The symptomatically stable patient is subjected to endoscopic, imaging and interventional angiographic investigations to determine the lesion balance and establish subsequent to the therapeutic conduct.

Depending on the symptomatology and clinical examination of the patients with cervical trauma selected for this study, the following methods of paraclinical exploration were used: cervical and thoracic X-ray, Doppler cervical and soft part ultrasound, craniocerebral, cervical and thoracic computed tomographic scan, computed tomographic angiography, naso-pharyngo-laryngeal and upper digestive endoscopic exploration.Cervical and chest X-rays identify cervical soft tissue emphysema, fractures, tracheal lacerations, foreign body retention, hemothorax, pneumothorax, mediastinal emphysema. Computed tomography, ultrasound of the cervical soft parts and Doppler, angiography, or pharyngoesophageal transit with contrast material are performed when complex lesions are suspected. Panendoscopic exploration (laryngeal, pharyngo-esophageal and tracheo-bronchoscopy) are used in the case of complex lesions of the aerodigestive axis. The use of the rigid endoscope is superior to the flexible one in upper esophageal lesions.

For patients with aero-digestive lesions depending on the severity of the symptoms and the injury complexity, the therapeutic attitude involved a medical treatment associated with the surgical one. Patients with mild laryngeal and tracheal injuries, without respiratory changes or signs of laryngeal fractures, were hospitalized and kept under observation for at least 24–48 h (since there is a potential risk of further respiratory dysfunction, through the expansion of edema, in the interval of time, which follows the trauma). The treatment was conservative, with medicinal support, and consisted of administering antibiotic therapy, steroid anti-inflammatories, aerosols, oxygen therapy, and air humidification. During hospitalization, it is indicated to carry out serial evaluations clinically and through naso-pharyngo-laryngeal fiberoscopy.

In the case of patients with variable respiratory disorders associated with laryngeal mucosa lesions, edema, and hematomas, the intervention was performed by performing a tracheotomy under local anesthesia and an endoscopic lesion assessment after the induction of general anesthesia. 3–5 days postoperatively, the tracheotomy was suppressed, during which the patient received supportive drug treatment. The presentation at admission with signs and different degrees of acute respiratory insufficiency associated with extensive laryngeal lesions of the mucosa, edema, and endolaryngeal hematomas, alteration of the mobility of the vocal cords or different degrees of comminution of cartilaginous fractures required the surgical therapeutic approach. This was performed in the following sequence: tracheotomy, endolarynx exploration through direct laryngoscopy, cervical wound exploration, and laryngeal approach through thyrotomy or the thyroid cartilage fracture path. Lesions of the laryngeal mucosa were repaired by sutures with absorbable 4-0 or 5-0 threads without exposing cartilaginous areas. Depending on each case’s lesion, the foot of the epiglottis, the ventricular band, or the vocal cords were reinserted, the aryepiglottic fold was restored, and the laryngeal fractures were reduced by suturing with non-absorbable threads. Anchorage of the superior and inferior retrocricoid ends was required. In cases with significant loss of substance, the larynx was restored with different muscles plasties. Anchorage of the hyoid with separate wires to the external laryngeal perichondrium was performed to achieve adequate laryngeal tightness and statics. Surgical therapeutic management was associated with antibiotic treatment, steroid anti-inflammatories, aerosols, and oxygen therapy. Suppression of the tracheal cannula was performed after 10–12 days.

The most severe cases with poor prognosis were characterized by extensive destructive lesions, loss of soft and cartilaginous substance, vocal cord disinsertion, or crico-tracheal disjunction associated with severe acute respiratory failure. These required emergency surgical exploration, laryngeal reconstruction, and recalibration. Recalibration was performed by fitting and maintaining a Montgomery tube for two weeks. Again, appropriate laryngeal calibration is recommended, with scar management in major debridement and mucosal restoration to avoid stenoses.

In cases with hypopharyngeal lesions of varying degrees of complexity, a careful clinical examination was performed, digestive rest was indicated with the application of a nasogastric tube, and depending on the severity of the lesion, surgical reconstruction as quickly as possible (ideally within the first 6–8 h from trauma) associated with performing a tracheotomy. The suturing of the pharyngeal wall was performed in two planes, serous and mucous. Hypopharyngoesophageal injuries are severe and require immediate recognition and treatment. A muscle flap can be used as a sleeve and isolates the upper digestive tract to avoid the formation of pharyngo-cutaneous or eso-tracheal fistulas. Pharyngeal plastic surgery was performed with prevertebral aponeurosis and subhyoid muscles. They are ideal for preventing post-operative suppurative or stenotic complications. Significant is the prompt correction of metabolic and hydro-electrolytic imbalances in the pre-, intra-, and post-operative periods, such as the administration of correct broad-spectrum antibiotic therapy, anti-inflammatories, and proton pump and H2 receptor inhibitors to prevent regional complications and sequelae. Maintaining the nasogastric feeding tube is indicated in cases where pharyngeal sutures were performed and in the remission of fistulas for 3–4 weeks.

Depending on the complexity of the traumatic aero-digestive injury, digestive rest through a nasogastric tube and respiratory rest through tracheotomy for 7–14 days are necessary conditions for resuming normal swallowing, breathing, and phonation. In addition, aspiration drainage is applied and maintained to avoid the formation of postoperative hematomas and seromas to smooth the tissue planes.

Cervical wounds involving various tissues and the cervical visceral aerodigestive axis require an established protocol for a multidisciplinary team approach. This team consists of the otolaryngologist, anesthesia and intensive care physician, emergency physician, and other specialties depending on the injuries associated with different body segments.

Vascular lesions in the zone I required collaboration with the thoracic surgery department, with the performance of mediastinotomy and thoracotomy to perform hemostasis. The identification following the radiological examination of hemothorax, pneumothorax, or pneumomediastinum required proper drainage of air and blood collections. In one case with plurivisceral involvement and basicervical venous vascular section, the thoracic duct was also involved, along with anterior mediastinal lesions in the left half. This case also involved collaboration with the thoracic surgeon. In zone III, the lesions required the performance of a median mandibulotomy to expose the parapharyngeal space to evacuate hematomas with an obstructive dyspneic character or to have access to the vasculo-nervous structures at the base of the skull. In the case of cases involving the submandibular gland, excision of the torn tissues, suturing of the glandular body, or total or partial excisions of it were necessary.

Cervical venous injuries (jugular vein and thyro-lingo-facial venous trunk) were resolved by ligation and adventitial sutures except when both internal jugular veins were involved when an attempt was made to restore the continuity of one of them. In the case of the carotid artery injury, it was important to fix it by adventitial sutures, end-to-end anastomosis, or by applying a graft with the help of vascular surgery colleagues (two cases).

In the case of complex cervical trauma produced by a closed or penetrating mechanism, which involved the thyroid, the following aspects were identified: expansive anterior cervical hematoma, compressive on the respiratory axis with phenomena of acute respiratory failure, which required its emergency drainage; cervical wounds with thyroid and laryngeal involvement with bleeding, which flood the respiratory tract, causing the exacerbation of respiratory failure phenomena and which required the performance of protective tracheotomy and the securing of the airway associated with the removal of crushed glandular tissues or different degrees of subtotal thyroidectomy. In addition, in 7 of the cases included in the study group, the glandular suture of the suture type with slow resorbable thread was performed.

The postoperative occurrence of fever, tachycardia, chest pain, widening of the radiographic mediastinum, subcutaneous emphysema, bleeding wound, the onset of neurological deficits, or the development of a hematoma requires imaging and surgical re-exploration.

All cervical aero-digestive traumas, regardless of complexity, were carefully recorded in terms of local examination, the evolution of symptoms and vital or biological constants, imaging and endoscopic examination, and exploratory and surgical reconstruction protocols.

The clinical management of complex cervical trauma has changed over time towards a conservative approach. Even so, the need for surgical exploration in patients with traumatic penetrating injuries of the aerodigestive tract or great vessels should be based on clinical elements and reliable indicators for indicating an open exploration.

The number of medical and medical-surgical specialties involved and the diagnostic assessment of patients with traumatic neck injuries requires developing and implementing a multidisciplinary institutional protocol with national applicability. In addition, it could support the ways of indicating paraclinical diagnostic procedures, such as flexible naso-pharyngo-laryngeal fiberoscopy, plain radiography, and esophagography, as well as computed tomography, but also the clear indications of surgical versus conservative treatment.

Since Ambroise Pare ligated both carotid arteries and the jugular vein of a soldier with polytrauma of the neck in 1552, the therapeutic approach to complex penetrating cervical trauma has been one of the controversial topics in trauma surgery. There has not yet been a consensus on this. Surgical exploration of all neck injuries beyond the platysma muscle significantly reduced mortality during World War II [3], but 89% of interventions did not identify deep visceral injuries [4]. In 1969, Monson et al. divided the cervical region into the three zones used in the management of trauma diagnostic protocol [5]. Legerwood et al. in 1980 and Narrod et al. in 1984 showed that the absence of severe clinical signs of hemodynamic instability in penetrating limb trauma quite clearly excludes arterial injuries, which require surgical treatment [6,7]. They extrapolated this reasoning to the diagnosis and treatment algorithm of cervical trauma with vascular involvement, revealing that some arterial lesions may be missed in the diagnosis and may result in potentially life-threatening vascular accidents.

In the 1990s, two studies focused on clinical examination to identify severe signs of vascular involvement in zone II cervical trauma and the need for an indication for reparative surgical exploration [8,9]. In addition, the clinical examination is more than 99% accurate in diagnosing these lesions with a false-negative rate comparable to angiography [9]. Furthermore, clinical examination is faster, less expensive, and involves few medical staff. Even so, it is less likely to be able to detect minor lesions such as intimal vascular irregularities, pseudoaneurysms, and arteriovenous fistulas compared to angiographic exploration. Most of these lesions are of no clinical significance [10]. Therefore, routine angiography’s additional costs and morbidity are difficult to justify.

Some studies even promote mandatory surgical exploration of all penetrating cervical wounds based on low morbidity and reduced hospital days. This approach has been described as cost-effective and characterized by higher accuracy than contrast-enhanced imaging studies [10].

For patients with high suspicion of penetrating laryngotracheal injury, detailed clinical examination is mandatory. Trauma of the upper aerodigestive tract is suspected in face of dysphonia or stridor, subcutaneous emphysema appearance, or blowing wound presence, and the main objective is to ensure and maintain the upper airway patency. In a retrospective study including 748 patients with this type of injury, only 11% of cases required immediate airway control, raising questions about the importance of conservative measures [11]. Surgical wound management is indicated if there is a significant change in the laryngotracheal anatomy. In such cases, orotracheal intubation may worsen the existing injuries; therefore, tracheostomy under local anesthesia is recommended, even at the site of the accident.

Metheny et al. believe that nasogastric tube placement and feeding is more effective than parenteral nutrition in most patients with critical aero-digestive traumatic injury because it preserves the integrity of the bowel and causes fewer infectious complications. However, nasogastric tube feeding has been associated with risks like other therapies. The most serious potential complication is the tracheobronchial aspiration of gastric contents, with the risk of developing a series of clinically silent microaspirations [12].

As we emphasized before, starting with WWII, the main recommendation was to surgically explore the neck for any traumatic lesion that involves the platysma [13]. Over the years, various research showed that this may be associated with a significant number morbidity. An article from the late 1970s showed that 56% of surgical neck explorations were non-therapeutic [14]. The modernization of imaging technologies made the selective approach of neck exploration more common. In this matter, an important study showed that 207 of 312 patients (66%) presenting with penetrating neck injuries were able to be managed conservatively, due to a thorough physical examination, along with angiography/esophageal exploration. Only one patient appeared to have an esophageal lesion missed on the initial examination and required repeat exploration [10]

Nowadays, the advantages of a selective nonoperative management for penetrating cervical trauma have been established, but there remain some circumstances when immediate operative exploration is mandatory, such as significant airway or major vascular injuries [10]. In 2011, Burgess et al. [15] reviewed the specialized literature regarding managing traumatic cervical injuries with a penetrating character and the institution of mandatory or selective surgical treatment.

In the case of our study, all cervical traumas beyond the platysma were surgically explored under general or local anesthesia with the identification and surgical reconstruction of the lesion. The indication of paraclinical imaging studies was influenced by the hemodynamic status and the need to secure the airway by orotracheal intubation or tracheostomy. In addition, complex aero-digestive lesions required digestive rest and the placement of a nasogastric tube, with the administration of food through this route or the association with the parenteral administration of the necessary nutrients. In cases of reduced complexity, conservative management and supportive drug treatment of traumatic and associated pathology were associated, as appropriate.

Early recognition and management of the complications of penetrating cervical wounds are essential in reducing the mortality and morbidity of these injuries. Prevention of these complications depends on the initial therapeutic actions of securing the airway by intubation or tracheostomy, prompt control of bleeding, protection of the head and neck, accurate and rapid lesion diagnosis, and surgical treatment according to the indication [16]. Additionally, in this study, 89.7% of selected patients were identified with uncomplicated healing, while in our study, the rate was 94.3%, close to other studies [16,17].

## 5. Conclusions

The first evaluation of complex cervical trauma is part of the standard Advanced Trauma Life Support (ATLS) protocol, an essential element on which the vital prognosis depends: securing the airways by orotracheal intubation or tracheostomy. The prehospital period should not be prolonged by performing the ATLS protocol and involves simultaneously balancing vital signs. These elements can dramatically change the patient’s prognosis, transforming him from dying to savable. An unstable hemodynamic status and an airway, which requires emergency stabilization, determined the performance of the lesion balance by performing emergency surgical exploration and involves multidisciplinary therapeutic management, followed by reconstructive surgical treatment at the same operative time or a second time. Optimizing a multidisciplinary emergency medical system will lead to improving global statistics regarding this pathology’s mortality and morbidity.

## Data Availability

Database is available, upon reasonable request, to the corresponding authors.
